# Effects of oxygen availability on mycobenthic communities of marine coastal sediments

**DOI:** 10.1038/s41598-023-42329-1

**Published:** 2023-09-14

**Authors:** Yanyan Yang, Carmen Alicia Rivera Pérez, Tim Richter-Heitmann, Rolf Nimzyk, Michael W. Friedrich, Marlis Reich

**Affiliations:** 1 Molecular Ecology Group, Faculty of Biology and Chemistry, University of Bremen, Bremen, Germany; 2 Biodiversity and Evolution of Plants, Institute of Biology and Environmental Sciences, Carl von Ossietzky University of Oldenburg, Oldenburg, Germany; 3 Microbial Ecophysiology Group, Faculty of Biology and Chemistry, University of Bremen, Bremen, Germany; 4 MARUM, Center for Marine Environmental Sciences, University of Bremen, Bremen, Germany

**Keywords:** Marine biology, Microbial ecology

## Abstract

In coastal marine sediments, oxygen availability varies greatly, and anoxic conditions can develop quickly over low spatial resolution. Although benthic fungi are important players in the marine carbon cycle, little is known about their adaptation to fluctuating availability of oxygen as terminal electron acceptor. Here, we study which part of a mycobenthic community from oxic coastal sediments can thrive under temporarily anoxic conditions. We test whether phylogeny or certain fungal traits promote plasticity in respect to changes in oxygen availability. Therefore, we incubated mycobenthos under oxic and anoxic conditions, performed ITS2 Illumina tag-sequencing and an additional meta-analysis on a literature survey. Half of all OTUs showed a plasticity towards changing oxygen availability and exhibited different strategies towards anoxic conditions, with rapid response within hours or a delayed one after several days. The strategy of dimorphism and facultative yeasts were significantly linked to OTU occurrence in anoxic conditions, while phylogeny and other traits had less effect. Our results suggest that different fungal niches are formed over the duration of prolonged anoxic conditions. The taxon-specific proliferation seems to be regulated by the fine-tuning of various traits and factors. It is essential to take these results into account when conducting conceptual work on the functionality of the marine benthos.

## Introduction

Microorganisms in marine sediments play an important role in the oceanic carbon cycle and food webs. Through their decomposition activities, they control the rate and yield of carbon turnover with consequences for the long-term carbon storage in marine sediments^1^. Fungi are part of the microbial benthos, called mycobenthos, that can metabolize similar amounts of carbon as bacteria^2^.

Oxygen availability is an important factor for microbial activity, as molecular oxygen is the thermodynamically most preferable terminal electron acceptor for respiration. Many microbes have evolved alternative metabolisms allowing to thrive in niches with low or no oxygen content^3^. Fungi exhibit the broadest metabolic and physiological diversity among eukaryotes^4^. This includes adaptation strategies at the organelle level such as reduced mitochondria or hydrogenosomes^5, 6^, diverse energy yielding pathways like fermentation, aerobic or anaerobic respiration^7^ and the use of alternative electron acceptors to oxygen^8^. These findings are based on physiological experiments mainly conducted on a few terrestrial fungal species. Even within the realm of community-level investigations, the number of studies with a focused lens on mycobenthos remains limited, particularly those that delve into the intricate relationship between oxygen concentration and mycobenthic diversity and composition^9–12^. However, an important gap in this area is the absence of studies scrutinizing the impact of varying oxygen concentrations on sediments originating from the same sample location. Noteworthy among these is the research conducted by Ortega-Arbulu et al*.*^12^, which centered on sediments from a lagoon distinguished by its high macrophyte density. As a consequence, the adaptability of marine mycobenthos to the influence of shifting oxygen availability levels on mycobenthic diversity remains a subject that lacks definitive conclusions. Additionally, the correlation between such adaptational responses and the functional role of mycobenthos remains insufficiently explored.

Mycobenthos in coastal sediments face special challenges given the strong dynamics related to oxygen availability^13, 14^. On the one side, organic input is high due to the proximity to the coast and high phytoplankton activity. This increases microbial respiration in the upper centimetres of the sediment leading to local anoxic conditions^15^. On the other side, numerous factors promote oxygen penetration into deeper sediment layers, such as pore water advection, resuspension processes, and bioturbation^16, 17^. As a result, oxygen availability can quickly change over local and temporal scale. It can be assumed that a large proportion of the fungal taxa inhabiting this area, exhibit adaptability to transitions between oxic and anoxic conditions. The aim of this study was to investigate (i) which proportion and taxa of such a natural community can thrive under anoxic conditions, and (ii) whether the adaptability is related to taxonomy, specific functional traits or temporal duration of anoxic conditions. For this purpose, mycobenthic communities were sampled from the oxic layers of sediments from the northern Wadden Sea, North Sea, and incubated under oxic and anoxic conditions. Diversity was analysed by sequencing the internal transcribed spacer 2 (ITS2) of the fungal ribosomal RNA (rRNA). To put the results from the incubation approach into a broader context, we further performed a meta-analysis on published mycobenthic sequence data from coastal marine sediments and compared them to the results of the incubation experiment.

## Results

### Facts on the datasets

The meta-analysis of available studies identified seven fungi specific ITS2-HTS datasets comprising 158 samples, 132 from oxic and 29 from anoxic sediments (Suppl Table S1). Furthermore, 28 studies were found that described physiological adaptation strategies of fungi to hypoxia/anoxia experimentally (Suppl Table S1; Suppl Fig. S1). All rarefaction curves of the main dataset and the two sub-datasets levelled off reaching a plateau indicating sufficient sequencing depth to capture most of the mycobenthic diversity (Suppl Fig. S2).

The combined dataset, which includes data generated from the incubation experiments in this study and data from publicly available datasets (meta-dataset), revealed a total of 11,376 fungal operational taxonomic units (OTUs) comprising 10,202,128 sequences. OTUs were classified into 14 fungal phyla, 44 classes, 121 orders, 306 families and 826 genera. Nearly half (46%) of the relative sequence abundance (51% of all OTUs) were made up by Ascomycota, followed by Basidiomycota with 24% (12% of all OTUs). The group of unidentified fungi comprised 29% of the relative sequence abundance (33% of all OTUs). Twelve phyla were further identified but all falling below the threshold of 1.5% (Suppl Fig. S3; see Suppl Table S2 for fully annotated OTU table, Suppl File S1 for representative sequence of each OTU as fasta-file).

### Mycobenthos in the incubation experiments

**Figure 1:**
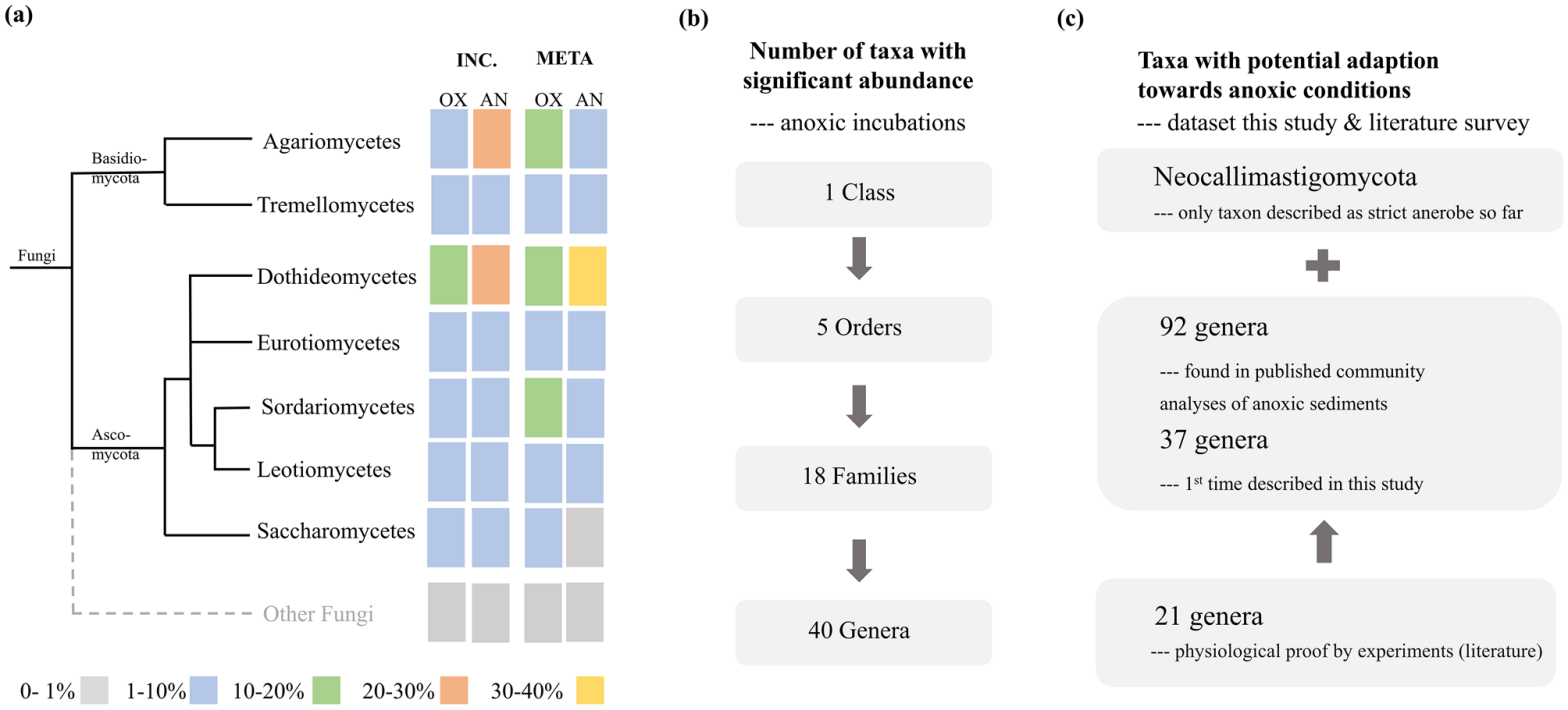
Fungal taxa in anoxic sediments. (**a**) Relative sequence abundance of the classifiable fungal taxa in oxic (OX) and anoxic (AN) sediments in the incubations (INC.) and meta-analysis (META). (**b**) Number of fungal taxa with a significant higher abundance in anoxic incubations over the different taxonomic levels (Tukey HSD, P < 0.05). (**c**) Number of fungal taxa that possess a potential adaptiveness/adaptation to anoxic conditions. Evidence based on literature survey including community analyses and physiological experiments. For the list of literature included see Supplementary Table S1.

**Figure 2:**
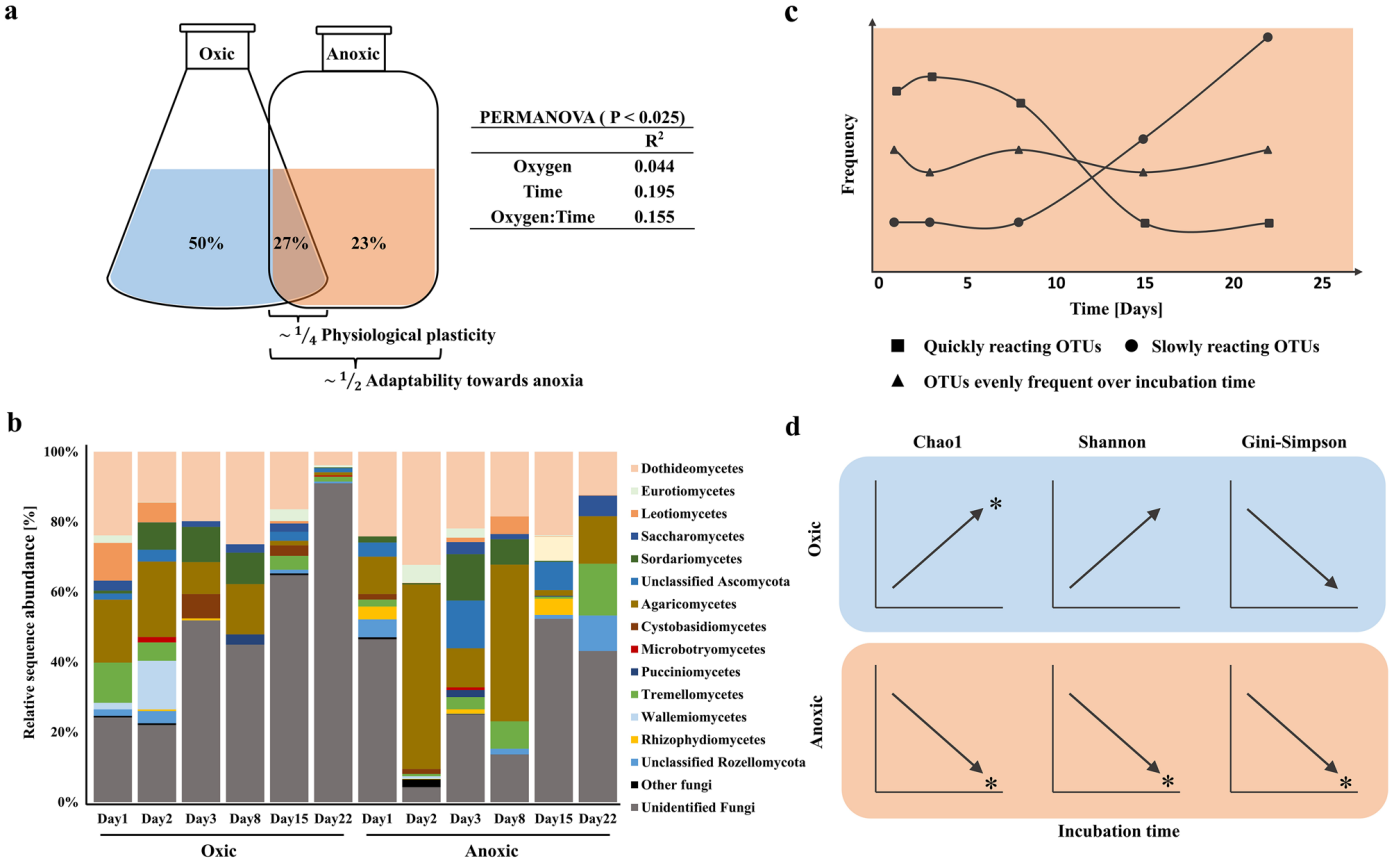
Alpha- and beta-diversity of mycobenthos in the incubation experiments. (**a**) All three tested factors, oxygen availability, time and oxygen availability*time, had a significant influence (PERMANOVA, P < 0.025) on beta-diversity. The incubation flasks show the percentage of OTUs detected in the different conditions. (**b**) Changes of the mycobenthic community over time in oxic and anoxic incubations. Only taxa that were represented in individual samples with > 1% relative abundance are shown. On higher taxonomic levels, only Rhizophyidomycetes showed significant difference between oxic and anoxic conditions (Tukey HSD test, P > 0.05). (**c**) OTUs detected in anoxic incubations were assigned to three reaction patterns over time since the start of incubation. (**d**) Alpha-diversity was mostly significantly influenced by incubation time and showed mostly opposite trends in oxic and anoxic incubations (linear regression, P < 0.05).

The incubation data revealed 614 OTUs comprising 928,656 sequences. Only 27% (165 OTUs) of all OTUs were detected in both conditions, while 23% (140 OTUs) of OTUs were found only in anoxic incubations (Fig. 2A). Ascomycota dominated both conditions with 22% (108 OTUs) and 35% (96 OTUs) of relative sequence abundance in the oxic and anoxic incubations, respectively. Basidiomycota accounted for 14% (53 OTUs) and 34% (55 OTUs) of the relative sequence abundance in the respective conditions. Rozellomycota accounted for 1% (8 OTUs) and 3% (5 OTUs), respectively. The percentage of unidentified fungi solely in aerobic incubations was high, accounting for 63% (304 OTUs) of the relative sequence abundance. Even with an additional manually BLASTn analysis against the UNITE database inspecting also potential species hypotheses (SH)^18^, the number could not be reduced. In anoxic incubations, 26% of the relative sequence abundance (146 OTUs) stayed unclassified. The remaining 2% of all reads in the communities consisted of members of eleven other phyla (Figs. 1A, 2B, Suppl Fig. S4).

At phylum level, no significant difference was found by the Tukey HSD test (P > 0.05) between oxic and anoxic incubations. However, from class level onwards, the number of significant different taxa increased with the taxonomic resolution, namely 1 class, 5 orders, 18 families and 40 genera (Fig. 1B, Suppl Table S3). Combining this result with information from the meta-dataset, the combined dataset held 92 genera with potential adaptive strategies to anoxia, 37 out of them described in the incubation experiment for the first time. For 21 genera out of the 92, experimental proof for their physiological adaptations towards hypoxia/anoxia exists according to literature (Fig. 1C, Suppl Table S1).

### Alpha- and beta-diversity of the mycobenthos in the incubation experiments

PERMANOVA revealed an effect of both, incubation time and oxygen availability, on beta-diversity (time: R^2^ = 0.195, P < 0.005; oxygen availability: R^2^ = 0.044, P < 0.005; interaction (oxygen availability*time): R^2^ = 0.155, P < 0.025) (Fig. 2A). The 305 OTUs found in anoxic incubations showed different temporal responses to anoxia. Out of them 199 OTUs increased in frequency over the first eight days of incubation. Out of these, 55 OTUs showed already a response within the first 24 h. A further 100 OTUs showed a delayed response to the new conditions and showed the greatest frequency increase after day 15. Six OTUs showed a relatively constant frequency (Fig. 2C).

For alpha-diversity, a significant time-dependent effect was identified by linear regression. OTU richness increased significantly in the oxic incubations (R^2^ = 0.61, P < 0.001) and decreased in the anoxic incubations (R^2^ = 0.24, P < 0.05) over time. Shannon diversity increased in oxic conditions and decreased in anoxic but only the latter one being significant (R^2^ = 0.30, P < 0.05). Evenness decreased in anoxic conditions significantly (R^2^ = 0.27, P < 0.05) (Fig. 2D; Suppl. Fig. S5). However, no significant differences were found when the two conditions regardless of time were compared (Tukey HSD). The Chao1 value was 65.1 ± 58.3 for the oxic and 42.3 ± 23.9 for the anoxic incubations. The Shannon values were 2.1 ± 0.5 and 2.3 ± 0.6. The Gini-Simpson index was the same in both conditions at 0.8 ± 0.1.

### Comparison of results from the incubation dataset with the findings from the meta-dataset

The meta-dataset held 11,038 OTUs comprising 9,273,472 sequences. As in the incubation approaches, Ascomycota dominated in both sediment types with 50% of the relative sequence abundance (5,560 OTUs) and 56% (1318 OTUs) in oxic and anoxic conditions, respectively. Basidiomycota accounted for 25% (1168 OTUs) and 19% (397 OTUs), respectively. The relative sequence abundance of unidentified fungi was considerably lower than in incubation experiments with 25% (3312 OTUs) and 25% (607 OTUs) in oxic and anoxic sediments, respectively. Twelve other phyla were neglectable with < 1.5% of relative sequence abundance (Fig. 1A, Suppl Fig. S6). The meta-dataset contained 45% (276 OTUs) of all OTUs that were also detected in the incubation dataset.

The number of OTUs with a physiological plasticity was lower in the meta-dataset than in incubations. Thus, only 15% (1659 OTUs) of all OTUs were found in both sediment types while 6% (699 OTUs) of OTUs were detected only in anoxic sediments (Table 1). The oxygen availability was a significant factor for the beta-diversity (PERMANOVA, F-value = 5.7, R^2^ = 0.03, FDR adjusted P < 0.001) as in incubation experiments. Similarly, alpha-diversity was significantly influenced (Tukey HSD, P < 0.001), with higher values in oxic sediments compared to anoxic sediments. The Chao1-values were 1015.5 ± 716.4 and 236.9 ± 101.7, while the Shannon-values were 3.7 ± 1.1 and 3.1 ± 1, respectively.

**Table 1:** Parameters that explain the significant differences between mycobenthos in oxic and anoxic sediments. The results of the incubation dataset and meta-dataset are listed separately.

	Incubation	Meta-analysis
Community characteristics		
Dominant phylum in anoxic sediment	Ascomycota	Ascomycota
Proportion of OTUs [%] occurring in anoxic/solely anoxic conditions [%]	50/23	21/6
Generalists	*Cladosporium*	*Cladosporium*
	*Alternaria*	*Alternaria*
		*Mortierella* …in total 25 taxa
Most contributing taxa via SIMPER with higher abundance in anoxic sediment: abundance [%]/COD^$^ [%]	*Lacrymaria* 9.6/6.25	*Psaytherella* 3.9/7.19
		*Alternaria* 4.4/3.88
Traits significantly related to OTU occurrence in anoxic sediments*	Dimorphic yeasts	Facultative yeasts
Traits significantly related to OTU occurrence in oxic sediments*		Facultative yeasts-microfungi
		Filamentous fungi
		Saprotrophy-symbiotrophy
Beta-diversity		
Significant factors^+^	Oxygen availability	Oxygen availability
	Incubation time	
	Oxygen availability: time	
Alpha-diversity (significant effects)		
OTU richness (Chao1)	Time-dependent increase in oxic sediment^§^	Higher in oxic sediment*
	Time-dependent decrease in anoxic sediment^§^	
Shannon diversity	Time-dependent decrease in anoxic sediment^§^	Higher in oxic sediment*
Gini-Simpson eveness	Time-dependent decrease in anoxic sediment^§^	

^$^contribution to overall dissimilarity

*Tukey HSD (P < 0.05); ^+^PERMANOVA (FDR-adjusted P < 0.05); ^§^linear regression (P < 0.025).

A total of 25 generalist taxa were identified in both the incubation dataset and the meta-dataset. However, only two generalist taxa, specifically *Alternaria* sp. and *Cladosporium* sp., were found in the incubation dataset, which also occurred in the meta-dataset (Suppl Table S4). SIMPER analysis (COD > 2%) identified one OTU as the most contributing taxon with higher abundance in the anoxic sediment for the incubations, namely *Lacrymaria* (10%, family Psathyrellaceae) and two OTUs in the meta-dataset, namely *Psathyrella* (4%) and *Alternaria* (4%) (Table 1, Suppl Table S5).

The trait analysis (Tukey HSD, P < 0.005) allowed the classification of 4089 OTUs. Two morphological traits were associated with significantly higher relative sequence abundance in anoxic sediments. In the incubation dataset, this was the growth form of dimorphic yeasts with 2.9% versus 4.3% in oxic and anoxic incubations, respectively. In the meta-dataset, facultative yeasts were significantly higher with namely, 1.3% in the oxic and 5.0% in the anoxic conditions. In contrast, the facultative yeasts-microfungi and the filamentous growth form were significantly more abundant under oxic conditions, with the relative sequence abundance of the facultative yeasts- microfungi reaching 2.7% under oxic conditions and only 1.3% under anoxic conditions. The filamentous form dominated with 86.7% compared to 78.2% under oxic and anoxic conditions, respectively. Of all trophic modi tested, only that of saprotrophy-symbiotrophy proved to be significantly different between oxic (5.8%) and anoxic (3.7%) conditions in the meta-dataset (Table 1, Suppl Table S6).

## Discussion

In this study, we examined the change in benthic fungal community composition and associated functional traits in a naturally occurring coastal surface sediment community subjected to both oxygenated (oxic) and oxygen-deprived (anoxic) conditions. Additionally, we performed a meta-analysis on publicly available datasets from previous studies, investigating mycobenthos diversity under different oxygen conditions. We hypothesised that due to the heterogeneity of oxygen availability in the source habitat, a large proportion of taxa possess the adaptability to thrive under (temporary) anoxic conditions. This priming effect was demonstrated for freshwater hyphomycetes originating from temporarily anoxic river sediment, which survived anoxic incubations compared to other fungal taxa^19^. Similarly, primed fungal communities from peat soils exhibited high levels of vital biomass in anoxic conditions in contrast to non-primed communities^20^. In our study, oxygen availability was a significant factor in both the meta-analysis and the incubation experiments. In the incubations, fifty percent of all OTUs were found under anoxic conditions, of which half were also detected under oxic conditions, representing a possible priming effect. The OTUs responded differently to anoxic conditions over time with some of them showing increased frequency within the first 24 h. In sediments, anoxic conditions can develop rapidly and already on a microscale level^21^. However, the mycobenthos in general seems to have a fast adaptability as shown by Ortega-Arbulu et al.^12^, in which a significant change to the slowly developing deoxygenation in the incubation bottles was detected after only 7 h. Our findings indicate that distinct fungal niches emerge within the sediment, both at the beginning and throughout the anoxic conditions. This leads to a time-resolved change in mycobenthic composition as observed here. Under natural conditions, the speed and sequence of temporal niche formation are certainly even more diverse as fungi possess the ability to respond with either high cell division activity^22^ or rapid spore germination within 1 h of activation^23^.

The significant occurrence of taxa under anoxic conditions increased at lower taxonomic levels, such as the family or genus level. At higher levels, Ascomycota and Basidiomycota dominated, but other phyla were represented. This suggests that the adaptability of marine mycobenthos in coastal sediments to (temporary) anoxic conditions is not phylogenetically clustered but rather phylogenetically dispersed. The underlying strategies and mechanisms may be diverse. For the entire fungal kingdom, only the Neocallimastigomycota have been described as obligate strict anaerobes so far. They live in the digestive tract of terrestrial and marine herbivores and possess specialised redox organelles called hydrogenosomes^7^. For the rest of the fungal kingdom, a wide range of physiological/metabolic mechanisms are known that allow fungi to thrive in (temporary) anoxic conditions^4^. This includes, among others, various fermentation and respiration processes using different electron donors as well as acceptors. Most physiological studies have been conducted on terrestrially isolated fungal strains^24–26^. Much less is known on marine-derived fungi. Some fungal isolates from temporarily anoxic coastal sediments showed nitrate reduction activity^27^. This strategy seems to be common among marine fungi. Manohar et al.^28^ identified several marine Ascomycota and Basidiomycota isolates as nitrate reducers in experiments simulating deep-sea, anoxic conditions. Furthermore, Stief et al.^29^ demonstrated ammonia fermentation and nitrous oxide production for an *Aspergillus terreus* strain isolated from the seasonal oxygen minimum zone of the Arabian Sea.

In the combined dataset of this study, numerous fungal genera were found for which physiological adaptation strategies to anoxic conditions were experimentally confirmed in respective studies. These taxa were represented in the oxic sediments in greater or at least the same frequency as in the anoxic sediments. From our study, however, it cannot be deduced to what extent the adaptation strategies for short, temporarily occurring anoxic conditions differ from those for life under permanent anoxic conditions. So far, marine fungal taxa from the Dikarya group are believed to have colonised the oceans in a secondary evolutionary step^30^. No significant phylogenetic signal related to anoxia has been found in marine fungal environmental data so far. Therefore, we would like to hypothesise that marine Dikarya fungi use older evolutionary adaptation strategies from their terrestrial time to thrive in marine anoxic conditions. Taxa from the basal fungal groups are excluded from this hypothesis. They largely diversified in aquatic systems^31^. Furthermore, it is assumed that their diversity is significantly greater than previously described^32^, and thus may have evolved metabolic and physiological mechanisms not yet identified.

In our study, taxa with different morphotypes were significantly associated with anoxic conditions, such as dimorphic yeasts. Dimorphic yeasts can reversibly change from a filamentous to a yeast growth form in the presence of environmental disturbances. In addition to oxygen availability, various factors such as temperature, pH or metabolites are cited as triggers for the morphological change^33^. The growth type under anoxic conditions can be variable, yeast-like or filamentous, and depends on the fungal species. However, a morphological change occurs through changes in expression and/or activation of specific genes, which includes genes for physiological activities, resulting in physiological changes of the organism^34^. Dimorphism, thus, gives fungal taxa an advantage to respond to environmental changes and to occupy different ecological niches.

Furthermore, facultative yeasts were also among the morphotypes significantly associated with anoxic conditions. This finding is consistent with the observation that most aquatic yeasts tested were described as weakly fermentative^35^. According to Fell and van Uden^36^, benthic yeasts are mainly found in the upper sediment centimetres. For most of them, decreasing oxygen concentrations is a limiting factor, as their synthesis of unsaturated fatty acids and sterols depends on the availability of oxygen^37^. Under temporary anoxic conditions, however, they can enter a fermentative mode. This allows them to compete in their specific niche. Interestingly, the facultative yeast group of Saccharomycetaceae have recently been described as active mycobenthos in sulphidic, permanently anoxic sediments^2^. It is speculated that the fermentative lifestyle of yeasts probably evolved through the exploration of anoxic niches, and as a result diverse yeast strains have since possessed growth under anoxic conditions^37^. Whether additional facultative yeast groups can flourish under consistently anoxic conditions can only be confidently ascertained through more extensive and taxonomically refined datasets. This lack of information may be one explanation, among others, for the contrasting depiction of the meta-dataset, wherein a significant prevalence of facultative yeasts is observed as opposed to facultative yeasts-microfungi in anoxic and oxic sediments, respectively.

The trait analysis further revealed abundance of fungi exhibiting mutlitrophic forms. Notably, the saprotrophic-symbiotic mode displayed significant prominence in the oxic sediment of the meta-dataset. The presence of a rich array of mixed trophic forms does not come as a surprise, given the profound eukaryotic diversity that characterizes the marine benthos across diverse kingdoms^38, 39^. Benthic fungi engage in multivarious interactions, encompassing diatoms^40, 41^, invertebrates, vertebrates^42^, bacteria^43^, and foraminifera^44^. Nonetheless, research endeavours concerning potential mutualistic interactions remain largely confined to investigations involving seagrass^45^ and corals^46^. Rojas-Jimenez et al.^47^ highlighted positive correlations observed among fungi within oxic deep-sea sediments. However, when interpreting trait data, it should be borne in mind that most of the information is based on data from terrestrial taxa. The marine environment presents fungi with very different challenges^48, 49^, which may lead to different trait expression.

Our findings of the combined datasets demonstrate that the adaptability of individual fungal taxa to anoxic conditions is probably controlled by various traits and factors. The regulation of whether, when and to what extent a fungal taxon proliferates presumably takes place via precise fine-tuning. However, the lack of studies on mycobenthos in general, and on its physiological adaptation to anoxic conditions in particular, hinders the development of a conceptual framework for its life in (temporary) anoxic sediments. Intensive studies are needed that should aspire to fill this gap using the latest standards of continuously evolving visualization and molecular tools. Thus, the benthic universe is open for mycologists to explore!

## Material and methods

### Incubation experiments

Five sediment cores were taken on the 11th of October in 2016 at low tide from the mudflats of the Wadden Sea of Dorum-Neufeld, Germany (53°44′12.5″N 8°30′29.3″E), using polycarbonate corers (25.5 cm length and 5.5 cm diameter) in a transect and with a distance of 5 m to each other. Corers were rinsed with 70% ethanol and flamed immediately prior to sampling. After sampling, the corers were secured with a rubber stopper at each side, placed in a cooler with ice, and transported in up-right position to the laboratory. In the laboratory, the top rubber stopper was replaced with Parafilm to allow oxygen exchange in the top layer and the corers were stored at 4 °C overnight. On the next day, the upper sediment layers (0–3 cm) from all cores were sliced off, homogenized, and used to prepare sediment slurry for incubations. Oxic incubations were set up in sterile 500-ml Erlenmeyer flasks, each containing an equivalent of 20 g of the mixed sediment and 60 ml of autoclaved artificial sea water (ASW; salinity of 35‰ mimicking North Sea water salinity)^50^, sealed with a cotton stopper and aluminum foil. Anoxic incubations were set up in sterile 120-ml serum bottles, each containing 20 g of mixed sediments and 60 ml ASW, sealed with a butyl rubber stopper. Dissolved oxygen was removed by three cycles of pulling vacuum for three minutes and flushing with N_2_ (99.999%; 0.5 bars). Three replicates were prepared per experimental setup. The microcosms were incubated at 15 °C in the dark. After 24 h, 48 h, 72 h, 8 days, 15 days, and 22 days, 2 ml of slurry were retrieved from each microcosm. Syringes were flushed with N_2_ before retrieving slurry samples from anoxic microcosms. All samples were stored at − 20 °C until further treatment.

### DNA extraction and Illumina sequencing on samples of the incubation experiment

DNA was extracted using the NucleoSpin® Soil Kit (Macherey–Nagel, Düren, Germany) according to the manufacturer’s instructions. The fungal ITS2 was amplified as described in Banos et al.^51^ but using the fungi-specific primers fITS7 (5′-GTGARTCATCGAATCTTTG-3′)^52^ and ITS4 (5′-TCCTCCGCTTATTGATATGC-3′)^53^. PCR, library preparations and sequencing were performed at LGC Genomics GmbH (Berlin, Germany) using the IlluminaMiseq chemistry for 2 × 300 bp reads (Illumina, Berlin, Germany) following the manufacturer’s instructions.

### Meta-analysis on publicly available fungal-specific ITS2 HTS data sets

The available literature was screened for High-Throughput Sequencing (HTS) datasets on mycobenthos from marine coastal sediment that targeted the ITS2, provided information on the oxygen content of the sampled sediment and were published by April 2022 at the latest. In short, a comprehensive literature research was conducted using search engines like “Web of Science” (https://www.webofscience.com/wos/woscc/basic-search), “Google Scholar” (https://scholar.google.com/), or the database of European Nucleotide Archive (ENA). Data was grouped into oxic or anoxic sediment according to contextual information in the papers. Additionally, papers and textbooks were screened for physiological proof of adaptation strategies of fungi towards hypoxia/anoxia.

### Sequence analysis and statistics

For comparability, all sequences from both the meta-dataset and the incubation dataset were analysed as one combined dataset using the PIPITS pipeline v3.0^54^, which uses the RDP classifier^55^ for taxonomic assignment. OTUs that were classified only to the kingdom level, were blasted against the UNITE^18^ and against the nucleotide INSD database (accessed 04.04.2022) using BLASTn^56^. For the different taxonomic levels, the sequence similarity thresholds were applied as in Tedersoo et al.^57^. To assign the trophic mode, and growth morphology to single OTUs, the FungalTrait datasheet v1.2^58^ was used.

To allow a more targeted statistics, additionally, two separate OTU tables were created based on the incubation dataset and- and the meta-dataset. If not differently stated, all analyses were carried out on the different OTU tables. Krona charts were generated on the taxonomic composition using KronaTools-2.8.1^59^. Generalists were defined by being present in more than 50% of all samples and occurring in oxic and anoxic conditions. All statistical analyses were carried out within the *R environment* v4.1.3^60^. Rarefaction curves were generated with the R package *iNEXT*^61^*.* OTU richness (Chao1), diversity (Shannon) and evenness (Gini-Simpson) was calculated using the “estimate_richness” function in the package *phyloseq*^62^. Impact of oxygenic conditions on taxa abundance over all taxonomic levels and alpha-diversity was calculated using Tukey’s post-hoc test (TukeyHSD) with an adjusted P-value. Significant occurrence of a given morphological trait or trophic modus under one condition was also calculated with the Tukey’s post-hoc test. On the incubation dataset, linear regression (n = 18; P < 0.05) was run additionally to identify a possible influence of the incubation time on the alpha-diversity using the “lm” function.

Next, the OTU-to-sample matrices were normalized via the total standardization using the “decostand” function in *vegan 2.6*^63^. Then, a Bray–Curtis dissimilarity matrix between pairs of samples was calculated. To identify genera/OTUs that contributed most to the observed dissimilarity between different conditions, the similarity percentage (SIMPER) analysis was done using the “simper” function in *vegan*. To test for significance of oxygen availability, time or both on beta-diversity PERMANOVA (FDR adjusted P < 0.05) was run using the “adonis2” function of the package *vegan.*

### Supplementary Information

Supplementary Legends.

Supplementary Figure S1.

Supplementary Figure S2.

Supplementary Figure S3.

Supplementary Figure S4.

Supplementary Figure S5.

Supplementary Figure S6.

Supplementary Information.

Supplementary Table S1.

Supplementary Table S2.

Supplementary Table S3.

Supplementary Table S4.

Supplementary Table S5.

Supplementary Table S6.

## Data Availability

The generated sequence datasets can be obtained from the European Nucleotide Archive (ENA) with the accession number PRJEB54572. The fully annotated OTU table can be accessed over the Supplementary Table S2, representative sequences for each OTU over the Supplementary File S1 (.fasta-format).
