# Supplementary material for: Effects of oxygen availability on mycobenthic communities of marine coastal sediments
Source: Sci Rep. 2023 Sep 14;13:15218. doi: 10.1038/s41598-023-42329-1 (PMC10502103; doi:10.1038/s41598-023-42329-1)
Supplement: Supplementary file 1 — Supplementary Legends. [file 41598_2023_42329_MOESM1_ESM.pdf]

Supplementary Material to "*Effects of oxygen availability on mycobenthic communities of marine coastal sediments*"

Yanyan Yang, Carmen Alicia Rivera Pérez, Tim Richter-Heitmann, Rolf Nimzyk, Michael W. Friedrich, Marlis Reich

Supplementary Tables:

Suppl Table S1: Information on samples of the incubation experiments and published ITS2-sequence data used for meta-analysis and literature survey (physiological experiments) (as .csv).

Suppl Table S2: Fully annotated OTU table (as .csv). Representative sequence for each OTU can be accessed via the Suppl File S1.fasta

Suppl Table S3: Taxa showing a significant difference in relative sequence abundance between oxic and anoxic incubations (Tukey HSD test, $P < 0.05$) (as .csv).

Suppl Table S4: Generalistic taxa in meta-analysis and incubation experiments (as .csv).

Suppl Table S5: SIMPER analyses on the sub-datasets of the meta-analysis and the incubation experiments. Only taxa with COD > 0. 5% were listed (as .csv).

Suppl Table S6: Tukey HSD test to identify traits related to a significant OTU abundance in anoxic sediment. Test carried out on the two subdatasets, namely incubations and meta-analysis (as .csv).

Supplementary Figures:

Suppl Fig S1: Overview of taxa with possible adaptability towards anoxic conditions arranged in a sketch of the fungal phylogenetic tree. Information is taken from this study, the meta-analysis and other literature showing experimental proof of adaptability. #, detected in anoxic incubations; *, significant difference in abundance between oxic and anoxic incubations; yellow, higher abundance in anoxic incubations; blue, proofed anoxic fermentation in literature survey; green, detected in anoxic

sediment of the meta-analysis; red, experimentally proofed anoxic fermentation and detected in anoxic sediment in literature survey; $, experimentally proofed anoxic respiration (as .jpg).

Suppl Fig S2: Rarefaction curves on oxic and anoxic sediment samples from (A, B) incubations and (C, D) meta-analysis (as .jpg).

Suppl Fig S3: Krona chart on the complete dataset containing information from samples of the incubation experiment and from the meta-analysis. Numbers of relative sequence abundance and OTU portion can be accessed separately (as .html).

Suppl Fig S4: Krona chart on the subdataset of incubation samples. Community composition in the oxic and anoxic condition can be accessed separately. Numbers indicate relative sequence abundance (as .html).

Suppl Fig S5: Linear regression ($n = 18$; $P < 0.05$) on alpha-diversity over incubation time in oxic (A-C) and anoxic (D-F) conditions (as .jpg).

Suppl Fig S6: Krona chart on the subdataset of meta-analysis samples. Community composition in the oxic and anoxic sediment can be accessed separately. Numbers indicate relative sequence abundance (as .html).

Supplementary File:

Suppl File S1: Representative sequence for each OTU (as .fasta).
