# Supplementary figures and images for: Effects of oxygen availability on mycobenthic communities of marine coastal sediments
Source: Sci Rep. 2023 Sep 14;13:15218. doi: 10.1038/s41598-023-42329-1 (PMC10502103; doi:10.1038/s41598-023-42329-1)

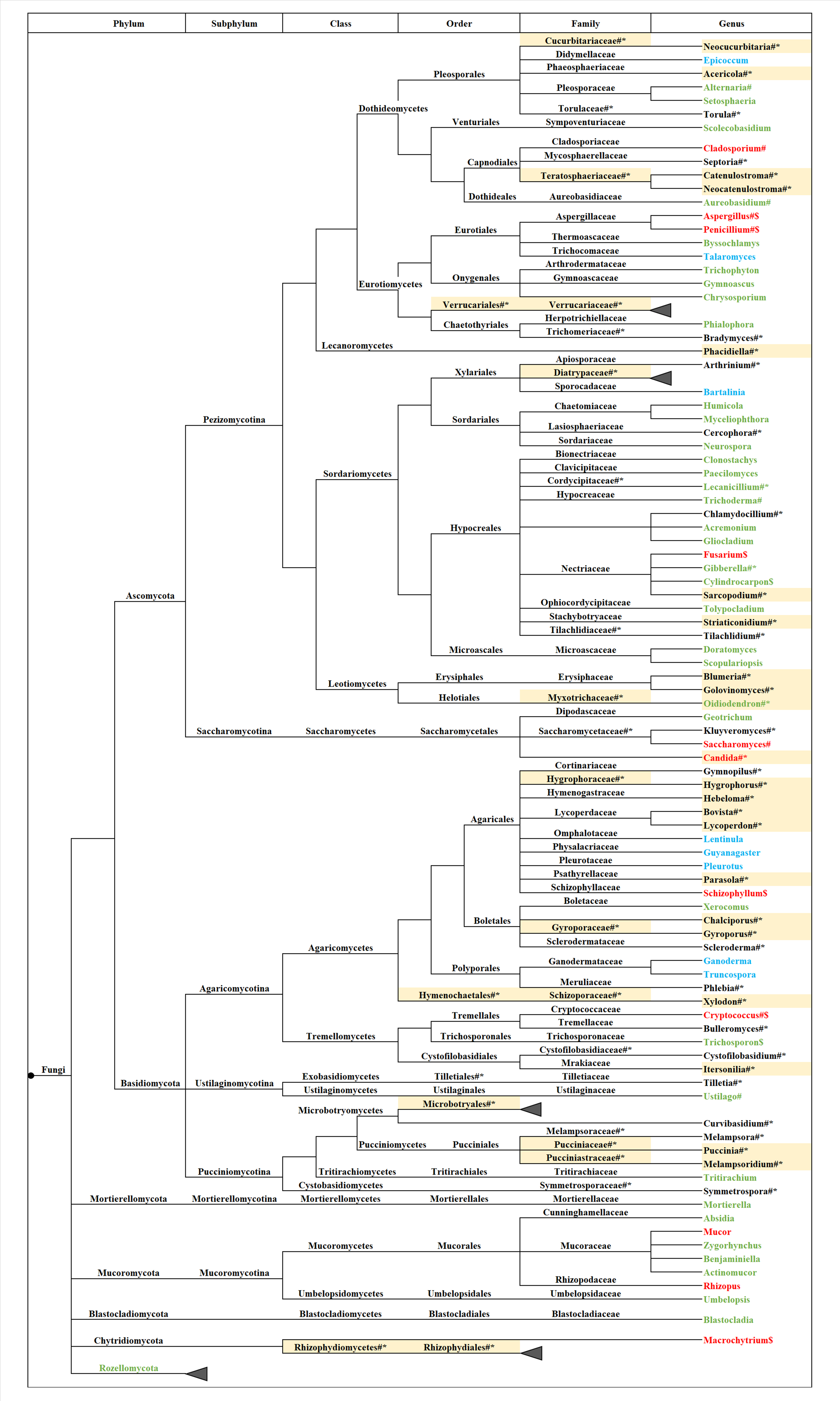

Supplement: Supplementary file 2 — Supplementary Figure S1. [file 41598_2023_42329_MOESM2_ESM.jpg]
