# Supplementary material for: Effects of oxygen availability on mycobenthic communities of marine coastal sediments
Source: Sci Rep. 2023 Sep 14;13:15218. doi: 10.1038/s41598-023-42329-1 (PMC10502103; doi:10.1038/s41598-023-42329-1)
Supplement: Supplementary file 4 — Supplementary Figure S3. [file 41598_2023_42329_MOESM4_ESM.html]

Javascript must be enabled to view this page.

magnitude

Sequences
OTUs

 10202128
 11376

 4732817
 5843

 72
 2

 72
 2

 72
 2

 72
 2

 988
 2

 988
 2

 988
 2

 988
 2

 2370238
 1791

 132
 5

 33
 1

 33
 1

 14186
 22

 1063
 2

 1063
 2

 6869
 14

 106
 1

 11
 1

 1565
 1

 439
 2

 2103
 2

 24
 1

 30
 1

 42
 1

 37
 1

 6254
 6

 2
 1

 466893
 191

 34
 2

 24
 1

 333444
 33

 287411
 15

 24784
 13

 1481
 3

 1413
 1

 2
 1

 66
 1

 31526
 5

 31526
 5

 21139
 37

 9
 1

 19
 1

 281
 1

 28
 1

 33
 1

 107
 1

 229
 1

 391
 3

 164
 3

 4
 1

 1963
 1

 17
 1

 13394
 1

 81
 2

 943
 3

 10521
 16

 10497
 14

 13648
 18

 42
 1

 1528
 1

 51
 2

 1433
 2

 50
 1

 10298
 2

 16
 1

 321091
 53

 155041
 24

 17665
 21

 1681
 6

 160
 2

 1472
 1

 19516
 9

 19516
 9

 17481
 6

 46
 1

 46
 1

 15
 2

 15
 2

 15
 2

 550
 3

 15
 1

 15
 1

 91
 5

 91
 5

 89
 4

 14
 1

 14
 1

 1420053
 1292

 14
 1

 14
 1

 9238
 9

 1797
 3

 7441
 6

 13
 1

 13
 1

 381
 3

 176
 1

 44
 1

 161
 1

 7604
 9

 7604
 9

 4012
 6

 3431
 4

 581
 2

 500
 3

 500
 3

 25991
 16

 59
 2

 10534
 1

 410
 6

 14958
 6

 22527
 22

 22272
 15

 102
 2

 8
 1

 25
 1

 35
 1

 351008
 81

 14
 1

 14
 1

 7144
 1

 6
 1

 81
 1

 29278
 14

 596
 2

 55
 1

 97
 2

 211795
 120

 84
 1

 2
 1

 134
 3

 16
 2

 4097
 4

 114
 1

 993
 1

 97402
 31

 61902
 31

 32258
 7

 79
 1

 3758
 5

 2066
 8

 1559
 1

 400
 2

 24
 1

 6
 1

 67
 2

 10
 1

 3741
 8

 2883
 4

 12
 2

 844
 1

 508
 1

 508
 1

 3403
 17

 10
 1

 67
 1

 697
 7

 6
 1

 4
 1

 4
 1

 8052
 16

 12
 1

 258
 5

 6472
 7

 120
 5

 16
 1

 6
 1

 69
 1

 20
 2

 20
 2

 20
 2

 15
 1

 14602
 28

 70
 1

 14532
 27

 106953
 91

 2083
 1

 4
 1

 283
 1

 54
 1

 139
 2

 5
 1

 14
 1

 1599
 5

 87
 3

 205
 4

 979
 15

 57
 1

 21
 1

 1084
 1

 372
 3

 99
 1

 117
 2

 23
 2

 5
 1

 267
 2

 101
 1

 101
 1

 248847
 106

 218039
 39

 1040
 1

 203
 2

 5999
 10

 83
 1

 3337
 5

 767
 3

 19
 1

 1030
 4

 24799
 61

 1754
 2

 54
 2

 3391
 20

 5
 1

 4
 1

 2280
 10

 3270
 17

 1414
 11

 2086
 2

 20
 1

 2066
 1

 171
 2

 36107
 37

 17
 1

 313
 2

 32726
 18

 2744
 10

 2744
 10

 15976
 11

 14770
 10

 1823
 2

 1823
 2

 78
 3

 78
 3

 61
 1

 138
 2

 138
 2

 138
 2

 23360
 41

 4150
 19

 113
 2

 2049
 9

 571
 1

 16096
 7

 17
 1

 15630
 1

 641991
 967

 69508
 219

 68
 2

 61
 1

 314
 11

 287
 10

 48607
 111

 45
 2

 1349
 12

 53
 3

 4759
 34

 17
 1

 40488
 10

 163
 3

 32
 1

 2764
 12

 1291
 4

 1473
 8

 530840
 539

 437624
 340

 210609
 87

 150
 3

 330
 1

 4
 1

 160567
 177

 267
 1

 97
 5

 31
 3

 66
 2

 51714
 105

 5
 1

 322
 3

 50882
 95

 43
 2

 6391
 113

 154
 3

 17
 1

 65
 3

 10
 2

 11
 2

 5
 1

 6
 1

 150
 1

 150
 1

 95
 12

 3
 1

 38
 4

 9
 1

 6
 1

 5
 1

 1615
 26

 273
 4

 6
 1

 13
 1

 230
 6

 27
 1

 550
 3

 44
 1

 44
 1

 945
 2

 945
 2

 41
 1

 350
 8

 350
 8

 294
 6

 135
 1

 79
 2

 80
 3

 4
 1

 4
 1

 4
 1

 4
 1

 63411
 49

 6
 1

 6
 1

 6
 1

 50526
 13

 11060
 5

 11060
 5

 39466
 8

 287
 2

 1068
 1

 310
 8

 5
 1

 5
 1

 23
 1

 23
 1

 15
 1

 15
 1

 47
 2

 4
 1

 218
 4

 162
 1

 162
 1

 56
 3

 43
 2

 7810
 6

 7810
 6

 393
 1

 2251
 1

 290
 1

 31
 1

 31
 1

 31
 1

 165726
 372

 52136
 24

 52136
 24

 11025
 3

 9
 1

 24526
 11

 114
 3

 16026
 1

 41
 2

 395
 3

 74709
 298

 19
 3

 2
 1

 17
 2

 2062
 29

 154
 1

 50
 2

 68
 3

 23
 1

 1163
 8

 439
 8

 6742
 18

 6141
 9

 9
 1

 174
 2

 11
 1

 61
 5

 52
 3

 9
 2

 2218
 44

 249
 1

 22
 4

 1897
 33

 8
 1

 48983
 21

 809
 4

 11
 1

 42
 1

 480
 4

 480
 4

 6
 1

 6
 1

 23
 3

 21
 2

 21
 2

 26252
 21

 26203
 18

 256
 2

 80
 2

 53
 1

 14752
 5

 5960
 4

 3
 1

 3
 1

 387
 17

 384
 16

 384
 16

 5
 1

 6
 1

 93
 6

 40
 1

 31789
 58

 31780
 57

 277
 6

 45
 1

 5719
 8

 423
 1

 1467
 4

 2505
 1

 5
 1

 5
 1

 22823
 9

 3
 1

 22394
 6

 818
 21

 5
 1

 76
 1

 49
 2

 128
 3

 10
 1

 357
 4

 273
 1

 3
 1

 3
 1

 102957
 135

 101944
 134

 21110
 7

 19781
 2

 44
 1

 20
 1

 1024
 1

 12
 1

 229
 1

 1997
 1

 1997
 1

 157
 6

 115
 5

 3454
 11

 3454
 11

 6600
 12

 11
 1

 4151
 2

 14
 1

 2420
 7

 642
 10

 160
 2

 482
 8

 25858
 27

 206
 7

 797
 3

 1643
 9

 22957
 2

 77
 1

 82
 3

 81
 1

 15
 1

 250
 3

 250
 3

 19
 2

 19
 2

 140
 3

 130
 2

 10
 1

 41
 1

 41
 1

 832396
 1613

 8
 1

 8
 1

 8
 1

 63
 2

 8
 1

 3553
 2

 3553
 2

 3553
 2

 1913
 23

 1913
 23

 375
 8

 325
 3

 196
 3

 7
 1

 2673
 41

 1498
 22

 513
 11

 401
 1

 19
 1

 19
 1

 19
 1

 119743
 83

 31011
 15

 31011
 15

 164
 6

 11
 1

 11
 1

 723
 2

 153
 1

 53361
 28

 147
 1

 2668
 1

 531
 10

 11800
 36

 12
 1

 12
 1

 2650
 12

 2520
 11

 9094
 21

 495
 1

 5950
 6

 3
 1

 181
 1

 53
 1

 2044
 5

 134
 2

 74
 1

 44
 2

 44
 2

 13
 1

 13
 1

 275676
 618

 9888
 31

 3
 1

 240
 6

 237
 3

 539
 1

 21
 1

 1908
 3

 4
 1

 57
 1

 10274
 34

 8384
 11

 110
 1

 177
 2

 73
 2

 1347
 9

 12
 2

 32
 1

 5671
 18

 423
 3

 365
 2

 166
 2

 1132
 4

 67
 1

 9
 1

 30186
 70

 27
 1

 45
 2

 207
 7

 29858
 58

 102003
 131

 56
 2

 1623
 1

 817
 2

 27
 1

 39
 2

 8356
 28

 2312
 7

 3409
 3

 65
 1

 27
 1

 17
 1

 2254
 3

 116
 6

 19
 1

 668
 2

 41
 1

 263
 2

 70
 1

 126
 5

 23
 1

 154
 5

 12
 2

 142
 3

 1824
 22

 13
 1

 8
 1

 607
 3

 4
 1

 3
 1

 747
 9

 23676
 71

 99
 3

 880
 8

 210
 4

 14
 1

 37
 1

 756
 2

 1462
 3

 229
 3

 134
 2

 7355
 5

 159
 2

 1110
 2

 262
 1

 768
 1

 768
 1

 10591
 13

 9885
 12

 133
 2

 562
 3

 9173
 5

 413
 18

 413
 18

 75
 1

 10
 3

 9
 1

 4
 1

 4
 1

 14
 2

 5
 1

 24654
 101

 83
 1

 83
 1

 137
 1

 137
 1

 9934
 38

 29
 1

 53
 1

 86
 3

 6132
 12

 2220
 11

 37
 1

 18
 1

 21
 1

 12990
 48

 569
 2

 312
 6

 95
 1

 98
 1

 1489
 12

 139
 4

 1488
 4

 94
 2

 37
 1

 166
 3

 166
 3

 166
 3

 518
 12

 514
 11

 7
 1

 2
 1

 410
 5

 24
 1

 24
 1

 24
 1

 18
 1

 18
 1

 18
 1

 75505
 213

 476
 7

 45
 3

 431
 4

 52812
 86

 3312
 15

 8
 1

 26
 1

 85
 4

 546
 6

 238
 1

 175
 2

 170
 1

 29
 1

 5
 1

 25
 2

 4
 1

 18
 1

 122
 5

 22
 2

 100
 3

 6704
 40

 321
 1

 19
 1

 387
 4

 48
 1

 621
 11

 579
 6

 102
 3

 97
 2

 333
 4

 333
 4

 333
 4

 4297
 6

 4297
 6

 4297
 6

 115009
 227

 4043
 14

 2878
 9

 18
 1

 18
 1

 42172
 35

 727
 2

 3981
 2

 15233
 9

 297
 3

 866
 4

 3526
 4

 57
 1

 3469
 3

 39435
 12

 35390
 9

 11010
 106

 728
 9

 563
 2

 5
 1

 20
 2

 232
 5

 75
 3

 9
 1

 57
 1

 5483
 19

 84
 2

 84
 1

 77
 2

 230
 1

 25
 1

 928
 1

 856
 22

 29
 2

 9335
 4

 9335
 4

 9335
 4

 9335
 4

 3
 1

 3
 1

 3
 1

 3
 1

 95
 1

 95
 1

 95
 1

 95
 1

 95
 1

 2459632
 1352

 1595504
 836

 953070
 400

 151406
 49

 84932
 21

 16866
 2

 3
 1

 10
 1

 8252
 7

 89
 2

 7
 1

 380
 1

 42
 6

 658
 3

 658
 3

 5034
 14

 28
 1

 172
 9

 12
 1

 400
 1

 668
 6

 38
 1

 572
 2

 1458
 9

 102
 3

 1350
 5

 1235
 8

 1195
 7

 40
 1

 178
 4

 46
 2

 133430
 22

 133141
 10

 266
 10

 25
 1

 25
 1

 690
 1

 690
 1

 2952
 6

 216
 1

 134
 3

 2602
 2

 731
 1

 731
 1

 696
 17

 442
 14

 3759
 24

 12
 1

 392
 5

 267
 5

 2346
 5

 3778
 4

 1208
 1

 117
 1

 87
 2

 39
 1

 18
 1

 18
 1

 289
 4

 284
 3

 622
 1

 622
 1

 118
 3

 104
 2

 42889
 8

 846
 6

 41978
 1

 357428
 68

 11203
 13

 3421
 19

 59205
 1

 2681
 5

 274774
 24

 1691
 2

 53
 1

 1638
 1

 3317
 1

 3317
 1

 124
 6

 15
 1

 5
 1

 40
 1

 8130
 28

 41
 1

 86
 2

 3413
 3

 1767
 4

 702
 5

 1334
 3

 250
 2

 1592
 21

 773
 3

 3
 1

 24
 1

 372
 6

 13
 1

 42
 2

 25
 1

 23
 1

 250
 2

 404
 1

 404
 1

 404
 1

 335
 12

 335
 12

 90
 3

 27
 1

 163
 5

 12
 1

 4879
 10

 40
 1

 40
 1

 243
 2

 243
 2

 4477
 3

 33
 1

 4428
 1

 3065
 16

 31
 2

 23
 1

 37
 2

 29
 1

 8
 1

 2010
 1

 2010
 1

 26
 2

 26
 2

 15
 2

 15
 2

 374
 2

 374
 2

 90
 2

 90
 2

 7370
 23

 11
 1

 11
 1

 866
 13

 43
 2

 16
 2

 26
 2

 26
 2

 7
 1

 12098
 5

 5552
 3

 1790
 1

 3712
 1

 50
 1

 6546
 2

 6546
 2

 778
 27

 778
 27

 743
 25

 69
 1

 69
 1

 69
 1

 3814
 38

 1246
 21

 999
 9

 24
 1

 116
 5

 19
 1

 31
 1

 3
 1

 2287
 12

 33
 2

 42
 1

 5
 1

 2020
 4

 8372
 20

 277
 3

 273
 2

 4
 1

 8085
 16

 66
 2

 31
 1

 6188
 9

 53323
 91

 788
 2

 783
 1

 5
 1

 5532
 10

 1678
 2

 568
 4

 3
 1

 146
 1

 73
 3

 73
 3

 3569
 4

 3233
 1

 336
 3

 12
 2

 10
 1

 12205
 19

 1126
 1

 873
 8

 3835
 6

 73
 3

 1248
 15

 59
 1

 828
 1

 248
 10

 12
 1

 64
 1

 37
 1

 175
 2

 114
 1

 61
 1

 26031
 14

 20239
 1

 3
 1

 1070
 1

 2333
 2

 1718
 6

 119
 3

 87
 2

 37
 1

 37
 1

 41747
 38

 1224
 1

 1224
 1

 53
 2

 48
 1

 31615
 13

 5854
 10

 8718
 19

 22
 2

 8677
 15

 12
 1

 12
 1

 2090
 22

 1595
 13

 86
 8

 109
 4

 39
 1

 508
 10

 508
 10

 357
 2

 28
 3

 2366
 34

 847
 21

 44
 1

 91
 2

 699
 17

 2048
 8

 2048
 8

 630
 2

 630
 2

 1366
 4

 450
 1

 98
 2

 16
 1

 16
 1

 16561
 48

 2251
 21

 2023
 20

 1924
 19

 99
 1

 658
 11

 439
 5

 109
 1

 330
 4

 10976
 27

 274
 2

 3310
 11

 321
 5

 321
 5

 44
 3

 39
 2

 5
 1

 2307
 2

 2094
 1

 638
 1

 638
 1

 15
 1

 15
 1

 15
 1

 7221
 10

 46
 1

 46
 1

 46
 1

 110
 2

 110
 2

 110
 2

 2403
 5

 2403
 5

 2403
 5

 2403
 5

 12864
 21

 12864
 21

 6404
 13

 6404
 13

 37180
 44

 460
 4

 460
 4

 460
 4

 116
 4

 102
 3

 102
 3

 398
 3

 64
 2

 53
 1

 34820
 22

 34820
 22

 4327
 2

 21756
 11

 7271
 5

 6072
 10

 18
 1

 18
 1

 1945
 3

 1025
 1

 1025
 1

 713
 1

 713
 1

 207
 1

 207
 1

 60
 3

 60
 3

 60
 3

 57
 3

 27
 2

 27
 2

 27
 2

 384490
 249

 3134
 11

 71
 4

 71
 4

 2964
 5

 674
 2

 2290
 3

 14152
 25

 12886
 20

 12681
 17

 185
 2

 20
 1

 1266
 5

 1266
 5

 1531
 7

 219255
 155

 15593
 2

 15593
 2

 88222
 64

 377
 2

 5821
 12

 40776
 9

 41248
 41

 21056
 15

 20998
 14

 58
 1

 75
 1

 75
 1

 19
 1

 19
 1

 5865
 13

 5865
 13

 39
 1

 39
 1

 8047
 6

 1474
 1

 6573
 5

 77
 1

 77
 1

 67230
 34

 67230
 34

 1172
 4

 19814
 19

 9674
 2

 60
 1

 3236
 33

 374
 2

 374
 2

 261
 1

 2862
 31

 2335
 29

 39
 2

 33
 1

 12
 1

 21
 2

 14
 1

 2200
 20

 14455
 13

 14455
 13

 14455
 13

 14455
 13

 13
 1

 28159
 103

 3071
 12

 17
 1

 17
 1

 17
 1

 61
 2

 88
 2

 88
 2

 32
 2

 32
 2

 13
 1

 13
 1

 19
 1

 19
 1

 10610
 13

 10610
 13

 1405
 2

 1405
 2

 367
 1

 367
 1

 200
 6

 200
 6

 12
 2

 12
 2

 168
 3

 49
 1

 26
 1

 157
 2

 102
 1

 102
 1

 102
 1

 102
 1

 482
 33

 16
 2

 16
 2

 2
 1

 410
 26

 40
 4

 40
 4

 3
 1

 370
 22

 280
 15

 219
 11

 74
 6

 17
 2

 2
 1

 8
 2

 8
 2

 40
 1

 40
 1

 7934
 66

 7934
 66

 7934
 66

 7919
 65

 6196
 57

 6427
 35

 57
 1

 57
 1

 5769
 20

 5769
 20

 2
 1

 2
 1

 38
 1

 38
 1

 178
 3

 89
 2

 89
 1

 679
 11

 581
 10

 16
 1

 16
 1

 4856
 3

 4856
 3

 601
 14

 28
 2

 573
 12

 573
 12

 573
 12

 644
 13

 644
 13

 644
 13

 644
 13

 28
 1

 292
 1

 292
 1

 292
 1

 292
 1

 292
 1

 38947
 169

 2926328
 3752

 161
 4

 154
 3

 154
 3
