# Supplementary material for: Effects of oxygen availability on mycobenthic communities of marine coastal sediments
Source: Sci Rep. 2023 Sep 14;13:15218. doi: 10.1038/s41598-023-42329-1 (PMC10502103; doi:10.1038/s41598-023-42329-1)
Supplement: Supplementary file 5 — Supplementary Figure S4. [file 41598_2023_42329_MOESM5_ESM.html]

Javascript must be enabled to view this page.

magnitude

Anoxic
Oxic

 357529
 571127

 124750
 123076

 0
 0

 0
 0

 0
 0

 0
 0

 0
 0

 0
 0

 0
 0

 0
 0

 83459
 73055

 0
 0

 0
 0

 0
 0

 0
 0

 0
 0

 0
 0

 0
 0

 0
 0

 0
 0

 0
 0

 0
 0

 0
 0

 0
 0

 0
 0

 0
 0

 0
 0

 0
 0

 0
 0

 28333
 11722

 0
 0

 0
 0

 18853
 9858

 18853
 9857

 0
 0

 0
 0

 0
 0

 0
 0

 0
 0

 86
 0

 86
 0

 120
 1861

 0
 0

 0
 0

 0
 0

 0
 0

 0
 0

 0
 0

 0
 0

 0
 0

 0
 0

 0
 0

 0
 1861

 0
 0

 0
 0

 0
 0

 26
 0

 0
 0

 0
 0

 9274
 1

 0
 0

 1528
 0

 0
 0

 0
 0

 0
 0

 7746
 1

 0
 0

 4054
 15902

 4045
 15883

 5
 25

 0
 0

 0
 0

 0
 0

 0
 0

 0
 0

 0
 0

 0
 0

 0
 0

 0
 0

 0
 0

 0
 0

 0
 0

 0
 0

 0
 0

 0
 0

 0
 0

 0
 0

 0
 0

 0
 0

 51061
 44638

 0
 0

 0
 0

 0
 0

 0
 0

 0
 0

 0
 0

 0
 0

 0
 0

 0
 0

 0
 0

 0
 0

 0
 0

 0
 0

 0
 0

 0
 0

 0
 0

 0
 0

 0
 0

 14841
 3750

 0
 0

 10519
 0

 0
 0

 4322
 3750

 0
 0

 0
 0

 0
 0

 0
 0

 0
 0

 0
 0

 7549
 17528

 0
 0

 0
 0

 0
 1

 0
 0

 0
 0

 1356
 1

 0
 0

 0
 0

 0
 0

 3474
 2193

 0
 0

 0
 0

 0
 0

 0
 0

 0
 0

 0
 0

 0
 0

 0
 0

 2441
 2193

 1033
 0

 0
 0

 0
 0

 0
 0

 0
 0

 0
 0

 0
 0

 0
 0

 0
 0

 0
 0

 0
 0

 0
 0

 0
 0

 0
 0

 0
 0

 0
 0

 0
 0

 0
 0

 0
 0

 0
 0

 0
 0

 0
 0

 0
 0

 0
 0

 0
 0

 0
 0

 0
 0

 0
 0

 0
 0

 0
 0

 0
 0

 0
 0

 0
 0

 0
 0

 0
 0

 0
 0

 0
 0

 0
 0

 2081
 2

 2081
 2

 0
 0

 0
 0

 0
 0

 0
 0

 0
 0

 0
 0

 0
 0

 0
 0

 0
 0

 0
 0

 0
 0

 0
 0

 0
 0

 0
 0

 0
 0

 0
 0

 0
 0

 0
 0

 0
 0

 0
 0

 0
 0

 7868
 6773

 5841
 6772

 0
 0

 0
 0

 0
 0

 0
 0

 0
 0

 0
 0

 0
 0

 0
 0

 2763
 0

 0
 0

 0
 0

 13
 0

 0
 0

 0
 0

 0
 0

 0
 0

 0
 0

 0
 0

 0
 0

 0
 0

 0
 0

 0
 0

 0
 0

 0
 0

 0
 0

 0
 1330

 0
 1330

 0
 0

 0
 0

 0
 0

 0
 0

 0
 0

 0
 0

 0
 0

 0
 0

 0
 0

 0
 0

 0
 0

 0
 0

 0
 0

 0
 0

 0
 0

 0
 0

 0
 0

 0
 0

 6246
 7804

 5056
 7756

 0
 0

 0
 0

 0
 0

 0
 0

 0
 0

 0
 0

 0
 0

 0
 0

 0
 0

 0
 0

 0
 0

 0
 0

 0
 0

 1
 1443

 0
 1006

 1
 437

 287
 6

 287
 6

 0
 6

 0
 0

 0
 0

 0
 0

 287
 0

 0
 0

 0
 0

 0
 0

 0
 0

 0
 0

 0
 0

 0
 0

 0
 0

 0
 0

 0
 41

 0
 0

 0
 0

 0
 0

 0
 0

 0
 0

 0
 0

 0
 0

 0
 0

 0
 0

 0
 0

 0
 0

 0
 0

 0
 0

 0
 0

 0
 0

 0
 0

 0
 0

 0
 0

 0
 0

 0
 0

 0
 0

 0
 0

 0
 0

 0
 0

 903
 1

 903
 1

 0
 0

 0
 0

 0
 0

 0
 0

 0
 0

 0
 0

 0
 0

 0
 0

 0
 0

 0
 0

 0
 0

 356
 0

 0
 0

 0
 0

 0
 0

 0
 0

 0
 0

 0
 0

 0
 0

 0
 0

 0
 0

 0
 0

 0
 0

 0
 0

 0
 0

 0
 0

 0
 0

 0
 0

 0
 0

 0
 0

 0
 0

 0
 0

 0
 0

 0
 0

 0
 0

 0
 0

 0
 0

 0
 0

 0
 0

 0
 0

 0
 0

 0
 0

 0
 0

 3991
 11544

 2739
 0

 2739
 0

 2693
 0

 0
 0

 0
 0

 46
 0

 0
 0

 0
 0

 0
 0

 738
 3067

 0
 0

 0
 0

 0
 0

 0
 0

 0
 0

 0
 0

 0
 0

 0
 0

 0
 0

 0
 0

 0
 0

 0
 0

 0
 0

 0
 0

 0
 0

 0
 0

 0
 0

 0
 0

 545
 0

 0
 0

 0
 0

 545
 0

 0
 0

 102
 3067

 0
 0

 0
 0

 0
 0

 0
 0

 0
 0

 0
 0

 0
 0

 0
 0

 0
 0

 0
 0

 2
 2711

 2
 2711

 0
 0

 0
 0

 0
 0

 2
 1648

 0
 0

 0
 0

 0
 0

 0
 0

 0
 0

 0
 0

 0
 0

 0
 0

 0
 0

 0
 0

 0
 0

 0
 0

 0
 0

 0
 0

 0
 0

 0
 0

 0
 0

 0
 0

 0
 0

 0
 0

 0
 0

 0
 0

 0
 0

 0
 0

 0
 0

 0
 0

 0
 0

 0
 0

 0
 0

 0
 0

 0
 0

 0
 0

 0
 0

 7815
 6473

 7815
 6473

 2382
 0

 2382
 0

 0
 0

 0
 0

 0
 0

 0
 0

 0
 0

 1143
 848

 1143
 848

 0
 0

 0
 0

 0
 0

 0
 0

 0
 2145

 0
 0

 0
 0

 0
 0

 0
 2145

 0
 0

 0
 0

 0
 0

 1
 2092

 0
 0

 0
 439

 1
 1273

 0
 380

 0
 0

 0
 0

 0
 0

 0
 0

 0
 0

 0
 0

 0
 0

 0
 0

 0
 0

 0
 0

 0
 0

 0
 0

 0
 0

 11783
 13297

 0
 0

 0
 0

 0
 0

 0
 0

 0
 0

 0
 0

 0
 0

 0
 0

 0
 0

 0
 0

 0
 0

 0
 0

 0
 0

 0
 0

 0
 0

 0
 0

 0
 0

 0
 0

 0
 0

 0
 0

 0
 0

 0
 1023

 0
 1023

 0
 1023

 0
 0

 0
 0

 0
 0

 0
 0

 0
 0

 0
 0

 0
 0

 0
 0

 0
 0

 0
 0

 0
 0

 0
 0

 0
 0

 0
 0

 0
 0

 0
 0

 0
 0

 0
 0

 0
 0

 0
 0

 0
 0

 0
 0

 0
 0

 0
 0

 0
 0

 0
 0

 0
 0

 4680
 9677

 0
 0

 0
 0

 0
 0

 0
 0

 0
 0

 0
 0

 0
 0

 0
 0

 0
 0

 1167
 0

 1167
 0

 0
 0

 0
 0

 0
 0

 0
 0

 0
 0

 0
 0

 0
 1039

 0
 0

 0
 0

 0
 0

 0
 1039

 0
 0

 0
 0

 1034
 1

 0
 0

 0
 0

 0
 0

 1034
 1

 937
 5006

 0
 0

 0
 0

 0
 0

 0
 0

 0
 0

 0
 0

 0
 0

 0
 2312

 0
 0

 0
 0

 0
 0

 0
 0

 0
 0

 0
 0

 0
 0

 0
 0

 0
 0

 0
 0

 0
 0

 0
 0

 0
 0

 0
 0

 0
 0

 0
 0

 0
 0

 0
 0

 0
 0

 0
 0

 0
 0

 0
 0

 611
 0

 0
 0

 0
 0

 0
 0

 0
 0

 0
 0

 0
 0

 0
 0

 0
 0

 0
 0

 0
 0

 0
 0

 611
 0

 0
 0

 0
 760

 0
 760

 0
 0

 0
 0

 0
 0

 0
 0

 0
 0

 0
 0

 0
 0

 0
 0

 0
 0

 0
 0

 0
 0

 0
 0

 0
 0

 0
 0

 0
 0

 0
 0

 0
 0

 0
 0

 0
 0

 0
 0

 0
 0

 0
 0

 0
 0

 0
 0

 0
 0

 0
 0

 0
 0

 0
 0

 0
 0

 0
 0

 0
 0

 0
 0

 0
 0

 0
 0

 0
 0

 0
 0

 0
 0

 0
 0

 0
 0

 0
 0

 0
 0

 0
 0

 0
 0

 0
 0

 0
 0

 0
 0

 0
 0

 0
 0

 0
 0

 0
 0

 0
 0

 0
 0

 3532
 308

 0
 0

 0
 0

 0
 0

 0
 0

 0
 0

 0
 0

 0
 0

 0
 0

 0
 0

 0
 0

 0
 0

 0
 0

 0
 0

 0
 0

 0
 0

 0
 0

 0
 0

 0
 0

 0
 0

 0
 0

 3532
 308

 0
 0

 0
 0

 0
 308

 0
 0

 0
 0

 0
 0

 0
 0

 0
 0

 0
 0

 0
 0

 0
 0

 0
 0

 0
 0

 0
 0

 3292
 2289

 0
 2289

 0
 2289

 0
 0

 0
 0

 3292
 0

 0
 0

 0
 0

 0
 0

 0
 0

 0
 0

 0
 0

 0
 0

 0
 0

 0
 0

 0
 0

 0
 0

 0
 0

 0
 0

 0
 0

 0
 0

 0
 0

 0
 0

 0
 0

 0
 0

 0
 0

 0
 0

 0
 0

 0
 0

 0
 0

 0
 0

 0
 0

 0
 0

 0
 0

 0
 706

 0
 706

 0
 706

 0
 706

 0
 0

 0
 0

 0
 0

 0
 0

 0
 0

 0
 0

 0
 0

 0
 0

 0
 0

 122089
 81666

 100157
 39057

 81873
 33903

 9528
 2390

 9528
 2390

 0
 0

 0
 0

 0
 0

 0
 0

 0
 0

 0
 0

 0
 0

 0
 0

 0
 0

 0
 0

 0
 0

 0
 0

 0
 0

 0
 0

 0
 0

 0
 0

 0
 0

 0
 0

 1
 1210

 0
 0

 1
 1210

 0
 0

 0
 0

 0
 0

 0
 0

 0
 0

 0
 0

 0
 0

 0
 0

 0
 0

 0
 0

 0
 0

 0
 0

 2145
 0

 0
 0

 0
 0

 2145
 0

 731
 0

 731
 0

 0
 0

 0
 0

 1978
 1

 0
 0

 311
 0

 0
 0

 1667
 1

 2446
 0

 0
 0

 0
 0

 0
 0

 0
 0

 0
 0

 0
 0

 0
 0

 0
 0

 0
 0

 0
 0

 0
 0

 0
 0

 0
 0

 0
 0

 0
 0

 39959
 25261

 1619
 0

 1550
 395

 34279
 24866

 2511
 0

 0
 0

 0
 0

 0
 0

 0
 0

 0
 0

 0
 0

 0
 0

 0
 0

 0
 0

 0
 0

 1906
 3828

 0
 0

 0
 0

 1906
 958

 0
 1636

 0
 0

 0
 1234

 0
 0

 0
 0

 0
 0

 0
 0

 0
 0

 0
 0

 0
 0

 0
 0

 0
 0

 0
 0

 0
 0

 0
 0

 0
 0

 0
 0

 0
 0

 0
 0

 0
 0

 0
 0

 0
 0

 0
 0

 0
 0

 0
 0

 0
 0

 0
 0

 0
 0

 0
 0

 0
 0

 0
 0

 2456
 343

 0
 0

 0
 0

 0
 0

 0
 0

 0
 0

 2010
 0

 2010
 0

 0
 0

 0
 0

 0
 0

 0
 0

 0
 343

 0
 343

 0
 0

 0
 0

 0
 0

 0
 0

 0
 0

 0
 0

 0
 0

 0
 0

 0
 0

 0
 0

 0
 0

 0
 0

 0
 0

 0
 0

 0
 0

 0
 0

 0
 0

 0
 0

 0
 0

 0
 0

 0
 0

 0
 0

 0
 0

 0
 0

 1891
 0

 0
 0

 0
 0

 0
 0

 0
 0

 0
 0

 0
 0

 0
 0

 1891
 0

 0
 0

 0
 0

 0
 0

 1891
 0

 675
 1811

 0
 0

 0
 0

 0
 0

 675
 1811

 0
 0

 0
 0

 675
 1811

 1285
 1169

 0
 0

 0
 0

 0
 0

 1274
 2

 0
 0

 0
 0

 0
 0

 0
 0

 0
 0

 0
 0

 0
 0

 0
 0

 0
 0

 0
 0

 0
 0

 0
 1167

 0
 0

 0
 0

 0
 1167

 0
 0

 11
 0

 11
 0

 0
 0

 0
 0

 0
 0

 0
 0

 0
 0

 0
 0

 0
 0

 0
 0

 0
 0

 0
 0

 0
 0

 0
 0

 0
 0

 0
 0

 0
 0

 0
 0

 0
 0

 0
 0

 2918
 0

 0
 0

 0
 0

 0
 0

 0
 0

 2918
 0

 0
 0

 0
 0

 0
 0

 0
 0

 0
 0

 0
 0

 0
 0

 0
 0

 0
 0

 0
 0

 0
 0

 0
 0

 0
 0

 0
 0

 0
 0

 0
 0

 0
 0

 0
 0

 0
 0

 0
 0

 0
 0

 0
 0

 0
 0

 0
 0

 0
 0

 0
 0

 0
 0

 0
 0

 0
 0

 2408
 6510

 17
 0

 17
 0

 17
 0

 0
 0

 0
 0

 0
 0

 0
 0

 0
 0

 0
 322

 0
 0

 0
 0

 0
 0

 0
 0

 0
 0

 0
 0

 0
 0

 0
 0

 0
 0

 0
 0

 0
 0

 0
 0

 0
 0

 0
 0

 0
 217

 0
 0

 0
 0

 0
 0

 0
 105

 0
 105

 0
 105

 0
 0

 0
 0

 0
 0

 0
 0

 994
 470

 994
 470

 0
 470

 0
 470

 334
 2086

 0
 0

 0
 0

 0
 0

 0
 0

 0
 0

 0
 0

 334
 0

 0
 0

 0
 0

 0
 889

 0
 889

 0
 0

 0
 0

 0
 889

 920
 1025

 0
 0

 0
 0

 920
 1025

 0
 1025

 0
 1025

 713
 0

 713
 0

 207
 0

 207
 0

 0
 0

 0
 0

 0
 0

 0
 0

 0
 0

 0
 0

 0
 0

 16457
 19635

 604
 1122

 0
 2

 0
 2

 537
 1120

 537
 0

 0
 1120

 0
 1624

 0
 1624

 0
 1624

 0
 0

 0
 0

 0
 0

 0
 0

 0
 0

 15048
 16186

 0
 874

 0
 874

 4193
 6723

 0
 0

 0
 0

 0
 0

 4193
 6723

 6453
 2452

 6453
 2452

 0
 0

 0
 0

 0
 0

 0
 0

 0
 0

 0
 0

 0
 0

 0
 0

 0
 0

 0
 1467

 0
 1467

 0
 0

 0
 0

 0
 0

 1
 703

 1
 703

 1
 703

 0
 0

 0
 0

 0
 0

 35
 0

 0
 0

 0
 0

 0
 0

 35
 0

 35
 0

 0
 0

 0
 0

 0
 0

 0
 0

 0
 0

 35
 0

 715
 12561

 715
 12561

 715
 12561

 715
 12561

 0
 0

 5876
 567

 378
 0

 0
 0

 0
 0

 0
 0

 0
 0

 0
 0

 0
 0

 0
 0

 0
 0

 0
 0

 0
 0

 0
 0

 0
 0

 4667
 567

 4667
 567

 0
 0

 0
 0

 0
 0

 0
 0

 0
 0

 0
 0

 0
 0

 0
 0

 0
 0

 0
 0

 0
 0

 0
 0

 0
 0

 0
 0

 0
 0

 0
 0

 0
 0

 0
 0

 0
 0

 0
 0

 0
 0

 0
 0

 0
 0

 0
 0

 0
 0

 0
 0

 0
 0

 0
 0

 0
 0

 0
 0

 0
 0

 0
 0

 0
 0

 0
 0

 0
 0

 0
 0

 0
 0

 0
 0

 0
 0

 0
 0

 0
 0

 0
 0

 0
 0

 0
 0

 0
 0

 0
 0

 0
 0

 0
 0

 0
 0

 0
 0

 0
 0

 0
 0

 0
 0

 0
 0

 0
 0

 0
 0

 0
 0

 0
 0

 0
 0

 0
 0

 0
 0

 0
 0

 0
 0

 0
 0

 0
 0

 0
 0

 0
 0

 0
 0

 0
 0

 0
 0

 0
 0

 0
 0

 10211
 6587

 94603
 359231

 0
 0

 0
 0

 0
 0
