# Supplementary material for: Effects of oxygen availability on mycobenthic communities of marine coastal sediments
Source: Sci Rep. 2023 Sep 14;13:15218. doi: 10.1038/s41598-023-42329-1 (PMC10502103; doi:10.1038/s41598-023-42329-1)
Supplement: Supplementary file 7 — Supplementary Figure S6. [file 41598_2023_42329_MOESM7_ESM.html]

Javascript must be enabled to view this page.

magnitude

Oxic
Anoxic

 6318159
 2955313

 2834750
 1650241

 72
 0

 72
 0

 72
 0

 72
 0

 117
 871

 117
 871

 117
 871

 117
 871

 1218556
 995168

 113
 19

 33
 0

 33
 0

 11245
 2941

 826
 237

 826
 237

 5281
 1588

 106
 0

 10
 1

 1
 1564

 439
 0

 2103
 0

 24
 0

 30
 0

 42
 0

 37
 0

 5138
 1116

 2
 0

 158400
 268438

 34
 0

 24
 0

 122945
 181788

 97926
 160775

 10795
 13989

 570
 911

 502
 911

 2
 0

 66
 0

 16
 31424

 16
 31424

 3077
 16081

 9
 0

 19
 0

 221
 60

 28
 0

 33
 0

 24
 83

 229
 0

 391
 0

 164
 0

 4
 0

 102
 0

 17
 0

 3
 13391

 81
 0

 7
 910

 1824
 8697

 1824
 8673

 1239
 3134

 42
 0

 0
 0

 51
 0

 591
 842

 50
 0

 319
 2232

 16
 0

 199449
 101686

 78102
 57011

 12413
 5222

 56
 1625

 6
 154

 1
 1471

 19513
 3

 19513
 3

 17478
 3

 0
 46

 0
 46

 15
 0

 15
 0

 15
 0

 39
 511

 15
 0

 15
 0

 91
 0

 91
 0

 89
 0

 14
 0

 14
 0

 718067
 606287

 14
 0

 14
 0

 1787
 7451

 1786
 11

 1
 7440

 13
 0

 13
 0

 381
 0

 176
 0

 44
 0

 161
 0

 7306
 298

 7306
 298

 3705
 307

 3431
 0

 274
 307

 500
 0

 500
 0

 6165
 1235

 59
 0

 15
 0

 410
 0

 5651
 1235

 21862
 665

 21659
 613

 102
 0

 8
 0

 25
 0

 0
 35

 73475
 252456

 14
 0

 14
 0

 306
 6837

 6
 0

 81
 0

 1041
 26880

 596
 0

 55
 0

 72
 25

 161356
 44772

 84
 0

 2
 0

 134
 0

 16
 0

 173
 3924

 114
 0

 990
 3

 88670
 8732

 49591
 7677

 8022
 23203

 79
 0

 3194
 564

 2061
 5

 1559
 0

 400
 0

 24
 0

 1
 5

 67
 0

 10
 0

 2887
 854

 2873
 10

 12
 0

 0
 844

 508
 0

 508
 0

 1253
 2150

 10
 0

 59
 8

 579
 118

 6
 0

 4
 0

 4
 0

 8041
 11

 12
 0

 249
 9

 6470
 2

 120
 0

 16
 0

 6
 0

 69
 0

 20
 0

 20
 0

 20
 0

 15
 0

 13051
 1551

 62
 8

 12989
 1543

 39599
 65271

 0
 0

 4
 0

 283
 0

 54
 0

 139
 0

 5
 0

 14
 0

 1599
 0

 0
 87

 205
 0

 933
 46

 57
 0

 21
 0

 2
 1082

 372
 0

 99
 0

 117
 0

 23
 0

 5
 0

 237
 30

 101
 0

 101
 0

 88832
 145374

 75099
 130327

 1019
 21

 203
 0

 4260
 1739

 83
 0

 2869
 468

 767
 0

 19
 0

 1029
 1

 20523
 1513

 1754
 0

 5
 49

 3307
 71

 5
 0

 4
 0

 2224
 56

 2842
 428

 1069
 345

 1914
 172

 20
 0

 1894
 172

 152
 19

 35202
 905

 17
 0

 313
 0

 31972
 754

 4
 1410

 4
 1410

 15790
 186

 14770
 0

 1656
 167

 1656
 167

 78
 0

 78
 0

 61
 0

 99
 39

 99
 39

 99
 39

 22175
 1185

 4134
 16

 111
 2

 2049
 0

 571
 0

 15863
 233

 17
 0

 15630
 0

 494759
 133182

 49447
 7249

 68
 0

 61
 0

 314
 0

 287
 0

 45312
 3295

 45
 0

 1332
 17

 53
 0

 4691
 68

 17
 0

 37417
 3071

 143
 20

 32
 0

 632
 688

 110
 175

 522
 513

 406084
 124463

 315866
 121465

 138685
 71918

 150
 0

 330
 0

 4
 0

 130813
 29467

 267
 0

 97
 0

 31
 0

 66
 0

 51281
 433

 5
 0

 322
 0

 50449
 433

 43
 0

 6015
 335

 154
 0

 17
 0

 65
 0

 10
 0

 11
 0

 5
 0

 6
 0

 150
 0

 150
 0

 95
 0

 3
 0

 38
 0

 9
 0

 6
 0

 5
 0

 1429
 186

 273
 0

 6
 0

 13
 0

 189
 41

 27
 0

 550
 0

 44
 0

 44
 0

 41
 0

 41
 0

 41
 0

 350
 0

 350
 0

 294
 0

 135
 0

 79
 0

 80
 0

 4
 0

 4
 0

 4
 0

 4
 0

 18162
 44893

 6
 0

 6
 0

 6
 0

 10964
 39562

 10733
 327

 10733
 327

 231
 39235

 210
 77

 0
 1068

 243
 67

 5
 0

 5
 0

 0
 23

 0
 23

 15
 0

 15
 0

 47
 0

 4
 0

 178
 40

 162
 0

 162
 0

 16
 40

 3
 40

 2678
 5132

 2678
 5132

 0
 393

 1204
 1047

 0
 290

 31
 0

 31
 0

 31
 0

 69656
 80535

 13364
 36033

 13364
 36033

 1599
 6733

 9
 0

 580
 23946

 68
 0

 10847
 5179

 41
 0

 220
 175

 26759
 44145

 19
 0

 2
 0

 17
 0

 1887
 175

 154
 0

 50
 0

 68
 0

 23
 0

 988
 175

 439
 0

 6742
 0

 6141
 0

 9
 0

 174
 0

 11
 0

 61
 0

 52
 0

 9
 0

 1669
 4

 249
 0

 22
 0

 1348
 4

 8
 0

 1951
 43863

 809
 0

 11
 0

 42
 0

 444
 36

 444
 36

 6
 0

 6
 0

 23
 0

 21
 0

 21
 0

 23349
 190

 23300
 190

 256
 0

 80
 0

 53
 0

 12912
 190

 5960
 0

 3
 0

 3
 0

 347
 40

 344
 40

 344
 40

 5
 0

 6
 0

 93
 0

 0
 40

 31306
 483

 31297
 483

 277
 0

 45
 0

 5618
 101

 423
 0

 1467
 0

 2467
 38

 5
 0

 5
 0

 22696
 127

 3
 0

 22394
 0

 817
 1

 5
 0

 76
 0

 49
 0

 128
 0

 10
 0

 302
 55

 273
 0

 3
 0

 3
 0

 71278
 17391

 70359
 17297

 2836
 15892

 1623
 15776

 44
 0

 20
 0

 1024
 0

 12
 0

 113
 116

 0
 6

 0
 6

 157
 0

 115
 0

 2960
 494

 2960
 494

 4440
 15

 11
 0

 4151
 0

 14
 0

 260
 15

 642
 0

 160
 0

 482
 0

 23723
 42

 206
 0

 321
 37

 369
 0

 22572
 5

 77
 0

 82
 0

 81
 0

 15
 0

 247
 3

 247
 3

 19
 0

 19
 0

 140
 0

 130
 0

 10
 0

 0
 41

 0
 41

 654275
 153041

 8
 0

 8
 0

 8
 0

 63
 0

 8
 0

 3553
 0

 3553
 0

 3553
 0

 1894
 19

 1894
 19

 374
 1

 307
 18

 196
 0

 7
 0

 2456
 217

 1304
 194

 465
 48

 396
 5

 19
 0

 19
 0

 19
 0

 107268
 11452

 29443
 545

 29443
 545

 164
 0

 0
 11

 0
 11

 723
 0

 153
 0

 47253
 6108

 137
 10

 186
 2482

 530
 1

 10973
 827

 12
 0

 12
 0

 2226
 424

 2096
 424

 8691
 403

 495
 0

 5825
 125

 3
 0

 181
 0

 53
 0

 1772
 272

 128
 6

 74
 0

 44
 0

 44
 0

 13
 0

 13
 0

 233946
 27373

 8080
 1808

 3
 0

 226
 14

 227
 10

 539
 0

 21
 0

 340
 1568

 4
 0

 57
 0

 1916
 7191

 99
 7118

 110
 0

 169
 8

 73
 0

 1314
 33

 12
 0

 0
 32

 4602
 30

 422
 1

 365
 0

 166
 0

 65
 28

 67
 0

 9
 0

 28913
 238

 27
 0

 45
 0

 207
 0

 28585
 238

 89776
 6284

 48
 8

 1
 1622

 817
 0

 27
 0

 39
 0

 8325
 31

 1658
 654

 2
 1095

 65
 0

 27
 0

 17
 0

 2254
 0

 116
 0

 19
 0

 610
 58

 41
 0

 255
 8

 70
 0

 123
 3

 0
 23

 154
 0

 12
 0

 142
 0

 1224
 600

 13
 0

 8
 0

 7
 600

 4
 0

 3
 0

 747
 0

 19623
 3442

 98
 1

 775
 105

 206
 4

 14
 0

 37
 0

 756
 0

 1211
 251

 225
 4

 76
 58

 6187
 1168

 159
 0

 499
 0

 262
 0

 8
 0

 8
 0

 6010
 4581

 5304
 4581

 95
 38

 550
 12

 4642
 4531

 408
 5

 408
 5

 75
 0

 10
 0

 4
 5

 4
 0

 4
 0

 14
 0

 5
 0

 21646
 3008

 83
 0

 83
 0

 131
 6

 131
 6

 6984
 2950

 29
 0

 53
 0

 86
 0

 3610
 2522

 1899
 321

 37
 0

 18
 0

 21
 0

 12938
 52

 569
 0

 312
 0

 92
 3

 98
 0

 1489
 0

 139
 0

 1439
 49

 94
 0

 37
 0

 166
 0

 166
 0

 166
 0

 507
 11

 503
 11

 7
 0

 2
 0

 399
 11

 24
 0

 24
 0

 24
 0

 18
 0

 18
 0

 18
 0

 69222
 2443

 476
 0

 45
 0

 431
 0

 52589
 223

 3312
 0

 8
 0

 26
 0

 85
 0

 417
 129

 238
 0

 175
 0

 170
 0

 9
 20

 5
 0

 25
 0

 4
 0

 18
 0

 102
 20

 22
 0

 80
 20

 2843
 21

 321
 0

 19
 0

 79
 0

 48
 0

 600
 21

 579
 0

 100
 2

 97
 0

 333
 0

 333
 0

 333
 0

 4251
 46

 4251
 46

 4251
 46

 83566
 25862

 1754
 0

 589
 0

 18
 0

 18
 0

 32162
 6718

 727
 0

 4
 3977

 12776
 2457

 296
 1

 757
 109

 3526
 0

 57
 0

 3469
 0

 27088
 12347

 23045
 12345

 5963
 5047

 728
 0

 68
 495

 5
 0

 20
 0

 232
 0

 75
 0

 9
 0

 56
 1

 1344
 4139

 71
 13

 0
 84

 32
 45

 230
 0

 0
 25

 928
 0

 752
 104

 29
 0

 1017
 7612

 1017
 7612

 1017
 7612

 1017
 7612

 3
 0

 3
 0

 3
 0

 3
 0

 95
 0

 95
 0

 95
 0

 95
 0

 95
 0

 1697943
 557934

 1257582
 198708

 712190
 125104

 139225
 263

 72822
 192

 16860
 6

 3
 0

 10
 0

 8239
 13

 89
 0

 7
 0

 328
 52

 42
 0

 7
 651

 7
 651

 5021
 13

 28
 0

 159
 13

 12
 0

 400
 0

 668
 0

 38
 0

 572
 0

 221
 26

 102
 0

 114
 25

 1235
 0

 1195
 0

 40
 0

 156
 22

 24
 22

 133338
 92

 133141
 0

 180
 86

 25
 0

 25
 0

 690
 0

 690
 0

 807
 0

 216
 0

 134
 0

 457
 0

 0
 0

 0
 0

 543
 153

 442
 0

 1694
 86

 0
 12

 81
 0

 254
 13

 678
 0

 1211
 121

 1088
 120

 117
 0

 87
 0

 39
 0

 0
 18

 0
 18

 289
 0

 284
 0

 0
 622

 0
 622

 118
 0

 104
 0

 42799
 90

 756
 90

 41978
 0

 175117
 117091

 9328
 256

 1474
 2

 60
 0

 170
 0

 160411
 114363

 0
 1691

 0
 53

 0
 1638

 626
 2691

 626
 2691

 124
 0

 15
 0

 5
 0

 40
 0

 2234
 162

 41
 0

 60
 26

 549
 0

 131
 0

 609
 93

 100
 0

 250
 0

 1592
 0

 773
 0

 3
 0

 24
 0

 372
 0

 13
 0

 42
 0

 25
 0

 23
 0

 250
 0

 404
 0

 404
 0

 404
 0

 335
 0

 335
 0

 90
 0

 27
 0

 163
 0

 12
 0

 418
 4461

 40
 0

 40
 0

 226
 17

 226
 17

 33
 4444

 33
 0

 0
 4428

 266
 0

 31
 0

 23
 0

 37
 0

 29
 0

 8
 0

 0
 0

 0
 0

 26
 0

 26
 0

 15
 0

 15
 0

 31
 0

 31
 0

 90
 0

 90
 0

 7370
 0

 11
 0

 11
 0

 866
 0

 43
 0

 16
 0

 26
 0

 26
 0

 7
 0

 3712
 8386

 3712
 1840

 0
 1790

 3712
 0

 0
 50

 0
 6546

 0
 6546

 559
 219

 559
 219

 545
 198

 69
 0

 69
 0

 69
 0

 1776
 147

 1099
 147

 856
 143

 24
 0

 116
 0

 19
 0

 31
 0

 3
 0

 396
 0

 33
 0

 42
 0

 5
 0

 129
 0

 5820
 66

 220
 57

 216
 57

 4
 0

 5590
 9

 58
 8

 31
 0

 3701
 1

 18480
 32389

 787
 1

 782
 1

 5
 0

 2366
 1890

 3
 1675

 353
 215

 3
 0

 146
 0

 73
 0

 73
 0

 333
 3236

 1
 3232

 332
 4

 12
 0

 10
 0

 9864
 1174

 0
 1126

 873
 0

 2668
 0

 27
 46

 376
 861

 48
 0

 0
 828

 215
 33

 12
 0

 64
 0

 37
 0

 175
 0

 114
 0

 61
 0

 838
 25193

 1
 20238

 3
 0

 0
 1070

 0
 2333

 166
 1552

 111
 8

 79
 8

 35
 2

 35
 2

 20855
 17974

 0
 1224

 0
 1224

 53
 0

 48
 0

 19623
 9074

 2334
 3520

 1134
 7584

 22
 0

 1093
 7584

 0
 12

 0
 12

 2051
 39

 1595
 0

 86
 0

 70
 39

 0
 39

 508
 0

 508
 0

 357
 0

 28
 0

 1026
 1340

 659
 188

 40
 4

 91
 0

 515
 184

 981
 1067

 981
 1067

 13
 617

 13
 617

 916
 450

 31
 419

 98
 0

 16
 0

 16
 0

 4942
 2701

 854
 1380

 851
 1155

 752
 1155

 99
 0

 639
 19

 420
 19

 109
 0

 311
 19

 1342
 9312

 0
 274

 1029
 2281

 321
 0

 321
 0

 44
 0

 39
 0

 5
 0

 242
 2065

 29
 2065

 422
 216

 422
 216

 15
 0

 15
 0

 15
 0

 247
 6757

 46
 0

 46
 0

 46
 0

 5
 0

 5
 0

 5
 0

 2403
 0

 2403
 0

 2403
 0

 2403
 0

 5923
 5477

 5923
 5477

 4336
 1598

 4336
 1598

 9764
 24996

 460
 0

 460
 0

 460
 0

 116
 0

 102
 0

 102
 0

 64
 0

 64
 0

 53
 0

 8935
 24996

 8935
 24996

 4327
 0

 4484
 17272

 64
 6318

 4127
 0

 18
 0

 18
 0

 0
 0

 0
 0

 0
 0

 0
 0

 0
 0

 0
 0

 0
 0

 60
 0

 60
 0

 60
 0

 35
 22

 5
 22

 5
 22

 5
 22

 215705
 132693

 1297
 111

 69
 0

 69
 0

 1196
 111

 26
 111

 1170
 0

 1566
 10962

 300
 10962

 95
 10962

 185
 0

 20
 0

 1266
 0

 1266
 0

 117
 1414

 125475
 62546

 4
 14715

 4
 14715

 67473
 9833

 377
 0

 4129
 1692

 40133
 643

 22834
 7498

 1830
 10321

 1772
 10321

 58
 0

 58
 17

 58
 17

 19
 0

 19
 0

 2727
 3138

 2727
 3138

 39
 0

 39
 0

 6523
 57

 7
 0

 6516
 57

 32
 45

 32
 45

 19307
 47219

 19307
 47219

 466
 2

 8052
 11762

 9673
 1

 60
 0

 1142
 2059

 374
 0

 374
 0

 261
 0

 768
 2059

 768
 1532

 36
 3

 21
 12

 12
 0

 21
 0

 14
 0

 648
 1517

 1175
 4

 1175
 4

 1175
 4

 1175
 4

 13
 0

 18753
 2963

 2399
 294

 17
 0

 17
 0

 17
 0

 61
 0

 88
 0

 88
 0

 32
 0

 32
 0

 13
 0

 13
 0

 19
 0

 19
 0

 5375
 1

 5375
 1

 1404
 1

 1404
 1

 367
 0

 367
 0

 200
 0

 200
 0

 12
 0

 12
 0

 168
 0

 49
 0

 26
 0

 157
 0

 102
 0

 102
 0

 102
 0

 102
 0

 482
 0

 16
 0

 16
 0

 2
 0

 410
 0

 40
 0

 40
 0

 3
 0

 370
 0

 280
 0

 219
 0

 74
 0

 17
 0

 2
 0

 8
 0

 8
 0

 40
 0

 40
 0

 7909
 25

 7909
 25

 7909
 25

 7894
 25

 6171
 25

 6416
 11

 57
 0

 57
 0

 5769
 0

 5769
 0

 2
 0

 2
 0

 38
 0

 38
 0

 178
 0

 89
 0

 89
 0

 679
 0

 581
 0

 16
 0

 16
 0

 4856
 0

 4856
 0

 590
 11

 28
 0

 562
 11

 562
 11

 562
 11

 436
 208

 436
 208

 436
 208

 436
 208

 0
 28

 292
 0

 292
 0

 292
 0

 292
 0

 292
 0

 21755
 394

 1728957
 743537

 161
 0

 154
 0

 154
 0
